# Nutritional Value, Health Properties, Safety Considerations, and Consumer Acceptance of *Lemnoideae* as Human Food

**DOI:** 10.3390/nu17183026

**Published:** 2025-09-22

**Authors:** Gabriela Zięć, Oskar Michalski, Anna Konieczna-Molenda, Tomasz Dera, Joanna Tkaczewska

**Affiliations:** 1 Department of Plant Products Technology and Nutrition Hygiene, Faculty of Food Technology, University of Agriculture in Kraków, Balicka 122, 30-149 Kraków, Poland; gabriela.ziec@urk.edu.pl (G.Z.); tomasz.dera@urk.edu.pl (T.D.); 2 Department of Chemistry, Faculty of Food Technology, University of Agriculture in Kraków, Balicka 122, 30-149 Kraków, Poland; oskar.michalski@urk.edu.pl (O.M.); anna.konieczna-molenda@urk.edu.pl (A.K.-M.); 3 Department of Animal Product Technology, Faculty of Food Technology, University of Agriculture in Kraków, Balicka 122, 30-149 Kraków, Poland

**Keywords:** *Wolffia globosa*, *Lemna minor*, water lentils, duckweed proteins, targeted nutrition, consumer perception, novel food regulation

## Abstract

Background: The growing demand for sustainable and nutrient-rich food has drawn attention to aquatic plants, particularly those of the *Lemnoideae* subfamily, commonly known as duckweed. These fast-growing plants are rich in high-quality protein and offer an attractive alternative to traditional plant and animal protein sources, especially in the context of targeted nutrition and sustainable diets. Methods: This review is a critical assessment of *Lemnoideae* potential as a functional food ingredient for various population groups, including vegans, diabetics, the elderly, and individuals with specific dietary needs. Their amino acid profile, protein digestibility, allergenicity, and bioactive compound content are evaluated in the paper. Also examined are health-related outcomes and applications, including clinical and space nutrition, alongside current barriers such as limited consumer acceptance and regulatory hurdles. Results and conclusions: *Lemnoideae* demonstrate a favorable amino acid profile, high digestibility, and low allergenic potential. They are a source of bioactive compounds with antioxidant, anti-inflammatory, and antimicrobial properties, and show promising effects in managing metabolic disorders such as type 2 diabetes and cardiovascular disease. Legal approvals in the EU and U.S. mark a shift towards broader acceptance. While sensory attributes and consumer unfamiliarity remain challenges, the findings allow us to highlight *Lemnoideae* as a promising next-generation plant protein source that could contribute to sustainable food systems and support the development of novel functional foods tailored to specific dietary needs.

## 1. Introduction

The growing global demand for sustainable and valuable food sources is driving the development of alternative food strategies, including interest in so-called “blue foods”—resources derived from aquatic environments, such as fish, algae, microalgae, and aquatic plants. In recent years, attention has been paid to aquatic plants, which are characterized by high protein content and a favorable amino acid profile. However, their broader application is limited by the low level of consumer acceptance, resulting from, among others, intense color and their characteristic, marine aftertaste [1,2].

Among aquatic plant organisms, freshwater species of the *Lemnoideae* (known as ‘water lentil’ and duckweed) subfamily are becoming of increasing interest as underestimated but promising sources of plant proteins. These plants exhibit rapid biomass growth, high nutritional value, and favorable sensory properties [1,3]. In Europe, protein consumption is still predominantly based on animal products—meat, dairy, fish, and seafood. According to data presented in the analysis by Prochazka, et al. [4], in the majority of European countries, the share of animal proteins significantly dominates over those from plants. This model of nutrition—based on a low share of plant proteins—is associated with poorer coverage of the demand for some dietary micronutrients. In accordance with the research results obtained by de la O, et al. [5], higher quality protein sources—including a greater share of plant proteins—correlate with a better supply of vitamins and minerals to the body.

The concept of so-called “targeted nutrition” is gaining increasing significance in nutritional sciences, an approach according to which adjusting diet composition to the specific needs of selected population groups is assumed. This applies not only to people with diseases such as type 2 diabetes, sarcopenia, or lipid disorders, but also to children, seniors, vegetarians/vegans, athletes, and patients with distinctive nutritional requirements. According to this approach, it is crucial to search for food raw materials that are characterized not only by high nutritional value, but also by good digestibility, low allergenicity, having bioactive ingredients, and potential health-promoting effects [6]. Plants from the *Lemnoideae* subfamily, due to their unique biochemical composition and functional properties, seem to fit perfectly into the assumptions of targeted nutrition (Table 1).

In several review papers, *Lemnoideae* have been addressed in recent years primarily with regard to their compositional profile, protein extraction methods, environmental and sustainability benefits, or food security potential [7,8,9,10]. However, in none of these works has a comprehensive assessment been provided of their role in targeted human nutrition, i.e., in the diets of specific population groups with distinct nutritional needs.

Despite their documented nutritional value, the potential of *Lemnoideae* remains underutilized, especially in the context of human nutrition with specific dietary needs [2]. There is a lack of reviews dedicated to the critical evaluation of not only their nutritional properties, but also aspects of consumer acceptance and safety, which are essential for successful implementation into human diets [11,12,13]. This represents a critical gap in the literature that we aim to address in our review.

An additional problem is the limited knowledge of the risks associated with the accumulation of heavy metals [11], microbiological contamination [12], and potential allergenicity of duckweed [13]. At the same time, as so-called “novel foods”, products derived from *Lemnoideae* are subject to rigorous legal regulations, which additionally require their thorough assessment before introducing them into broad circulation [1].

Consequently, the present manuscript provides an integrative review of the *Lemnoideae*, with a particular focus on their application in targeted nutrition for specific population groups (vegan, diabetic, elderly, inpatients, and space travelers). A particular emphasis is placed on issues of consumer acceptance and safety, which have been scarcely explored in previous reviews. By highlighting these aspects, our work offers a novel perspective that complements existing publications and underscores the unique role of *Lemnoideae* in the future of human nutrition.

**Table 1:** Application potential of *Lemnoideae* in targeted nutrition for specific population groups.

Population Group	Specific Nutritional Needs	*Lemnoideae* Contribution to Nutritional Needs	Knowledge Gaps/Research Needs	References
Vegans/vegetarians	Vitamin B12, complete protein, iron	*Wolffia globosa* provides bioavailable vitamin B12; *Lemnoideae* protein has a full amino acid profile and good iron bioavailability	Long-term clinical trials confirming safety and efficacy	[3,14,15]
Elderly (risk of sarcopenia)	High-quality protein, branched-chain amino acids (BCAA), good digestibility	*Lemnoideae* contain high levels of BCAA; protein digestibility ~89%, comparable to whey protein	Randomized controlled trials in elderly populations	[16,17,18]
Diabetics	Moderate postprandial glycemia, insulin response, glucose absorption and uptake, normalization of glycemia	Duckweed peptides have antidiabetic effects Positive glucose and insulin response after consuming *Lemna minor* Beneficial effects of *Wolffia globosa* on the gut microbiota and metabolism in the context of glucose metabolism	Broader human studies beyond pilot interventions	[19,20]
People with cardiovascular disease	Omega-3 fatty acids, antioxidants, fiber	Balanced *n*6/*n*3 ratio; presence of antioxidants (ferulic acid, luteolin, kaempferol) supports vascular health	Clinical validation of cardioprotective effects	[15,19,21]
Inpatients and space travelers	Compact, nutrient-dense source; rapid biomass growth; suitability for controlled cultivation	*Lemnoideae* species thrive in closed systems, provide sustainable protein, vitamins, and minerals, and are approved as GRAS and novel foods	Crop optimization and processing improvement for targeted applications	[9,16,22,23]

## 2. Literature Search Methodology

To retrieve the relevant literature, an integrative review combining a structured search and a critical analysis was performed. The literature search was performed between March and July 2025 in the following scientific databases: Web of Science, Scopus, ScienceDirect, and PubMed. The primary keywords included: *“Lemnoideae”*, “duckweed”, “water lentils”, *“Wolffia”*, “alternative protein”, “aquatic plants”, “functional food”, “targeted nutrition”, and related terms. Search strings were formed by combining keywords with Boolean operators.

The inclusion criteria were limited to research articles and publications in the English language. Publications such as books, book chapters, theses, and editorials were excluded to ensure high scientific quality and relevance. After the initial screening, detailed content analysis was conducted to prioritize articles that provided empirical data, theoretical insights, or significant contributions to the fields of food science, nutrition, and the application of *Lemnoideae* in human diets.

While this work does not follow the strict methodology of a systematic review, the adopted approach was designed to ensure a comprehensive and representative overview of the available literature, with a focus on the potential of *Lemnoideae* in targeted nutrition for specific population groups.

## 3. Plants of the *Lemnoideae* Subfamily: Taxonomic Characteristics, Nutritional Composition and Applications in Human and Animal Nutrition

The species diversity of the *Lemnoideae* family, its common occurrence at all latitudes, and its high nutritional as well as utility value show its enormous potential. For a long time, the plant known as duckweed was classified as the *Lemnaceae* family, with five genera (*Wolffia*, *Wolffiella*, *Lemna*, *Spirodela*, and *Landoltia*) [24]. However, botanists now propose an alternative taxonomy, according to which the *Araceae* Juss. family is divided into the *Lemnoideae* subfamily, including four genera: *Wolffia Horkel* ex Schleid, *Wolffiella* Hegelm., *Lemna* L., and *Spirodela* Schleid. The previously distinguished monotypic species *Landoltia punctata* has been included in this approach to the *Spirodela* genus and occurs under the name *Spirodela punctata*, and both species’ names are treated as synonyms. In this work, the taxonomy was adopted in accordance with the World Flora Online and Plants of the World Online (POWO) taxonomic databases.

All species of the *Lemnoideae* subfamily comprise freshwater plants that live near the surface of standing water or that with a very slow flow. These plants are distinguished by their small size and simplified structure. All species within this subfamily are capable of sexual reproduction, but they rarely produce flowers, and reproduction occurs almost exclusively in a vegetative manner by producing a daughter leaf that is easily separated. The dominant share of vegetative reproduction in these plants allows long-term cultivation of clones with desired utility features. The vegetative reproduction and growth of these plants are very fast, thanks to which their biomass can be doubled in favorable conditions within one to three days [25,26]. The percentage content of proteins, lipids, carbohydrates, and ash in the examined *Lemnoideae* species is summarized in Table 2. Since almost all *Lemnoideae* plants are composed of metabolizing tissues containing chloroplasts, they are characterized by high protein content and relatively low lipid and carbohydrate contents. Depending on the species, clone, culture conditions, and assay methodology, significant variation in the percentage of components is observed. Protein content is usually within the range of 20–30% dry mass, however, due to the selection of appropriate clones and optimization of culture conditions, their contents can even exceed 40% of dry mass. In unfavorable growth conditions, a decrease in protein contents is observed along with a simultaneous increase in starch contents [27]. *Lemnoideae* plants are rich in essential protein amino acids, and the protein found in the highest amounts is RuBisCo—a key enzyme involved in photosynthesis. In *Lemnoideae* plants, RuBisCo constitutes approximately 50% of the proteome [25]. The greatly diverse lipid and fat contents result not only from differences between plant materials, but also from different quantitative determination methods, and even different understandings of the terms “lipid” and “fat” by the authors of the publication. Similarly, the authors define “carbohydrates” differently, hence the very divergent results regarding the contents of these compounds in the examined plants. A more detailed discussion of the amino acid, carbohydrate, and lipid profile present in duckweed is available in the review by Takacs et al. [1] and in the publications cited therein.

**Table 2:** Taxonomy of *Lemnoideae* and its proximate composition.

Genus	Species	Protein [%]	Lipid/Fatty Acid/Fat [%]	Carbohydrate/Iber/Tarch [%]	Ash [%]	References
*Wolffia* Horkel ex Schleid	*W. angusta* Landolt	26	3.5 (f) 8.0 (fa)	16 (s)	13	[25,28,29]
	*W. arrhiza* (L.) Horkel ex Wimm.	17–51	0.5–6.1 (f) 8.9 (fa) 9 (lp)	2.4–15 (fb) 2.4–17 (s) 9.4–44 (c)	12–25	[25,27,29,30,31,32,33,34,35,36]
	*W. australiana* (Benth.) Hartog and Plas	28	2.0 (f) 10 (fa)	13 (s)	21	[25,29]
	*W. borealis* (Engelm.) Landolt	21–29	2.5 (f) 14 (fa)	11–14 (s)	16	[25,29,32]
	*W. brasiliensis* Wedd.	25	5.2 (f) 5.6 (fa)	11 (s)	19	[25,29]
	*W. columbiana* H.Karst.	23–36	0.7–6.6 (f)	11 (f) 12 (s)	17–22	[25,37]
	*W. cylindracea* Hegelm.	22	2.1 (f) 6.7 (fa)	11 (s)	22	[25,29]
	*W. elongata* Landolt	22	5.3 (f)	14 (s)	20	[25]
	*W. globosa* (Roxb.) Hartog and Plas	21–48	0.5–4.9 (lp) 2.1–9.6 (f) 11 (fa)	8.4–21 (fb) 10–29 (s) 37–48 (c)	7.7–24	[25,28,29,32,38,39,40,41,42,43,44]
	*W. microscopica* (griff.) Kurz	28–29	2.2–6.1 (f)	4.8–10 (s)	16–21	[25,45]
	*W. neglecta* Landolt	21	4.7 (f) 12 (fa)	14 (s)	12	[25,29]
*Wolffiella* Hegelm.	*W. caudata* Landolt	35	7.0 (fa)	9.7 (s)	n.d.	[29,32]
	*W. gladiata* (Hegelm.) Hegelm.	29	n.d.	8.2 (s)	n.d.	[32]
	*W. hyalina* (Delile) Monod	34	6.6 (f) 10 (fa)	5.8 (s)	n.d.	[29,45]
	*W. lingulata* (Hegelm.) Hegelm.	n.d.	5.7 (fa)	n.d.	n.d.	[29]
	*W. neotropica* Landolt	26	7.1 (fa)	10 (s)	n.d.	[29,32]
	*W. oblonga* (Phil.) Hegelm.	n.d.	10 (fa)	n.d.	n.d.	[29]
	*W. repanda* (Hegelm.) Monod	n.d.	7.6 (fa)	n.d.	n.d.	[29]
	*W. rotunda* Landolt	n.d.	n.d.	n.d.	n.d.	n.d.
	*W. welwitschii* (Hegelm.) Monod	n.d.	4.6 (fa)	n.d.	n.d.	[29]
*Lemna* L.	*L. aequinoctialis* Welw.	n.d.	9.3 (fa)	n.d.	n.d.	[29]
	*L. bistrosa* Charit.	n.d.	n.d.	n.d.	n.d.	n.d.
	*L. disperma* Hegelm.	n.d.	11 (fa)	n.d.	n.d.	[29]
	*L. gibba* L.	25–34	3.4–4.7 (f) 11 (fa)	1.9–14 (s) 3.3 (c) 9.4–26 (fb)	3.3–18	[29,32,37,45,46]
	*L. japonica* Landolt	26–36	8.0 (fa)	1.7–35 (s)	n.d.	[29,32,47,48]
	*L. landoltii* Halder and Venu	n.d.	n.d.	n.d.	n.d.	
	*L. minor* L.	7.5–42	1.3–4.7 (f) 1.5–9.0 (lp) 11 (fa)	1.7–48 (s) 8.2–27 (fb) 34–61 (c)	10–23	[29,32,45,47,49,50,51,52,53,54,55,56,57,58,59,60]
	*L. minuta* Kunth	26	1.0 (lp)	1.1–37 (s)	24	[47,61]
	*L. obscura* (Austin) Daubs	26–37	6.5 (lp) 7.5 (fa)	3.8 (s) 12 (fb)	5.6	[29,32,62]
	*L. perpusilla* Torr.	n.d.	5.5 (fa)	n.d.	n.d.	[29]
	*L. tenera* Kurz	n.d.	6.7 (fa)	n.d.	n.d.	[29]
	*L. trisulca* L.	n.d.	5.9 (fa)	n.d.	n.d.	[29]
	*L. turionifera* Landolt	n.d.	8.3 (fa)	2.5–32 (s)	n.d.	[29,47]
	*L. valdiviana* Phil.	24–29	1.0 (lp) 8.0 (fa)	5.6 (s)	2	[29,32,61]
*Spirodela* Schleid.	*S. oligorrhiza* (Kurz) Hegelm.	n.d.	n.d.	21 (s)	n.d.	[52]
	*S. polyrhiza* (L.) Schleid.	24–36	1.7–4.7 (f) 2.2–7.2 (lp) 7.5 (fa)	1.7–7.1 (s) 8.8–15.6 (fb) 38–42 (c)	9.7–21	[29,32,37,45,62,63,64,65,66]
	*S. punctata* (G.Mey.) C.H.Thomps.	14–38	3.8–5.5 (f) 6 (lp) 7.8–8.4 (fa)	1.1–25 (s) 9.2 (fb)	14	[29,32,34,37,45,67]
	*S. sichuanensis* M.G.Liu and K.M.Xie	n.d.	n.d.	n.d.	n.d.	n.d.

Abbreviations: (f)—fat; (fa)—fatty acid; (lp)—lipid; (s)—starch; (fb)—fiber; (c)—carbohydrate; n.d.—no data available.

Within the *Wolffia* Horkel ex Schleid genus, 11 species are recognized. They are the smallest known vascular plants. Their size does not exceed 1.5 mm. Their body is extremely simplified to one frond, spherical, ovoid, or boat-shaped, without roots. Almost the entire body of the plant comprises metabolizing tissue. Individuals occur singly or in pairs. In unfavorable conditions, they can form turions (spores) that sink to the bottom. The most common species of this genus is the cosmopolitan *W. arrhiza* (L.) Horkel ex Wimm., which reaches a size of 1 mm, and the slightly smaller (0.8 mm) *W. globosa* (Roxb.) Hartog and Plas, which occurs in Southeast Asia. Both species are used as additives to human food and as animal feed [25]. Wolffia plants are characterized by a very high protein content, usually 20–30% in dry matter, depending on the species, strain, and cultivation conditions. Detailed studies on the amino acid profile of these plants have allowed us to demonstrate that their proteins are rich in all essential amino acids in the diet and are close to the FAO recommendations from 2013. In numerous studies, the amino acid profile has been investigated [41,63,68]. Boonarsa et al. [41] reported that Wolffia is a valuable source of amino acids; however, its profile may not be complete for human nutrition. Therefore, it may be advisable to supplement its protein with other sources to ensure a balanced intake [41]. At the same time, the relatively low contents of fat (up to 6%) and starch (below 20%) means that *Wolffia* can be a valuable component of a low-calorie diet [25,31]. Moreover, many authors studied the fatty acid content of *Wolffia* [69,70]. *Wolffia* do not produce raphides, which are crystalline forms of calcium oxalate. The oxalate content of *W. arrhiza* has been shown to be low; thus, its consumption does not pose a risk of diseases caused by excess oxalates in the diet [31]. The *Wolffiella* Hegelm. genus includes 10 known species naturally occurring in Africa and the Americas. They are very small plants, about 2 to 10 mm in size. The body is simplified to an elongated frond without roots. *Wolffiella* are usually connected in easily separable clusters of several individuals. They do not form turions and are also more difficult to cultivate than *Wolffia* because they do not tolerate difficult environmental conditions. *Wolffiella* species are not yet used as a raw material for food production. In studies to date, it has been indicated that in terms of nutritional content, these plants are similar to *Wolffia*; the protein content is greatly comparable, and they do not form calcium oxalate crystals either [45].

Plants of almost all species from the *Lemna* genus grow near the water surface and produce a single root. The exception is *L. trisulca*, which has no root and spends most of its life submerged up to 2 m below the water surface. The fronds of plants from the *Lemna* genus are up to 5 mm in size, often forming large aggregations of connected individuals. Some species of *Lemna* produce raphides [71,72]. The crystalline form and amount of raphides may negatively affect the taste and health properties of plants in the context of their use as food or feed. Several *Lemna* species are intensively examined as high-protein feed additives: *L. gibba, L. minor, L. perpusilla, L. minuta,* and *L. obscura* [62,73]. In general, *Lemna* is characterized by a similar protein content to *Wollfia* and *Wolffiella*, but with an even lower starch content.

There are four known species belonging to the *Spirodela* genus. These are plants with the most diverse tissue structure within the *Lemnoideae* subfamily. A single frond is round or oblong, 3–10 mm long and up to 8 mm wide. These plants form clusters of up to 10 individuals connected to one another, each being composed of a single frond and several roots. A special feature of *Spirodela* is the reddish color of its lower surface. In unfavorable conditions, it can produce turions. All *Spirodelas* produce raphides in their cells [74]. The best-known species is the cosmopolitan *S. polyrhiza*. It is the largest plant in the *Lemnoideae* subfamily, reaching up to 15 mm of frond length and several centimeters of roots. Due to its easy cultivation and very fast growth, it is used as a protein supplement to feed [75,76].

Of particular importance are plants from the *Wolffia* genus, which are distinguished not only by the abundance of full-value protein, but also by low fat and starch content. The contents of exogenous amino acids in duckweed protein meet nutritional standards, and the presence of antioxidant compounds additionally increases its attractiveness [77]. These plants are already successfully used in the feed industry, supplements, and as components of the human diet, as well as ingredients in the formulation of human foods such as “Mankai”, snack bars, roll bread, and different bakery products fortified by duckweed proteins and other products [78,79]. All this makes *Lemnoideae* promising food resources that can be used in the nutrition of various population groups—from children, on to adults and seniors, or populations requiring specialist dietary support.

## 4. Food Safety and Legal Regulations Regarding the Use of *Lemnoideae*

For several years now, plants from the *Lemnoideae* subfamily have been considered healthy and affordable plant components of future foods and sustainable alternative sources of protein for animal meat replacement [80,81]. The two main species consumed by humans—*W. arrhiza* and *W. globosa*—known for their traditional Thai name, Khai-nam, have long been widely considered food for the poor. Duckweed was traditionally used to prepare dishes such as salads, omelets, or vegetable curries in some Southeast Asian countries (Burma, Laos, and northern Thailand) [9,25].

In a study on *Wolffia* consumption in Thailand (Loei Province), it was shown that locals still consume it in the traditional way, as an ingredient of spicy soups and salads [78,82]. Due to the fact that duckweed, and especially *Wolffie*, has a history of consumption in many areas, it is widely considered a “traditional third world food”. Traditionally, *Wolffia* plants are cultivated in local aquatic farms in Myanmar, Laos, and Thailand, where they are harvested twice a week from November to July. They are grown in open ponds.

The Thai government has provided documentation confirming the use of *Wolffia* spp., including the *W. arrhiza* and *W. globosa* species, as food that has been consumed by humans in the northern and northeastern parts of Thailand for generations [83]. Based on this evidence, *Wolffia globose* was submitted for authorization as a traditional food from a third country under the EU novel food regulation. The requester in this process was GreenOnyx Ltd. (Israel)**,** which included the Thai government documentation as proof of traditional consumption. *W. arrhiza* and *W. globosa* are also listed as edible vegetables in several databases (e.g., Local Vegetable of Thailand, United States Department of Agriculture GRIN database, Plants for the Future).

### 4.1. Legal Status of Duckweed as a Food Ingredient in Different Countries (European Union, USA, Asia)

The legal status of duckweed as a food ingredient varies across the world. In the European Union, the growing interest in plant-based diets has led to a reassessment of its value. At the request of the European Commission, the EFSA Panel on Nutrition, Novel Foods and Food Allergens (NDA) has issued several opinions on different *Lemnoideae*-derived products under Regulation (EU) 2015/2283. These include evaluations of *Wolffia globosa* powder [EFSA NDA Panel, 2021], water lentil powder from *Lemnaceae* [EFSA NDA Panel, 2021], whole plant material of *Lemna minor* and *Lemna gibba* [EFSA NDA Panel, 2022], and a water lentil protein concentrate from a mixture of *Lemna gibba* and *Lemna minor* [EFSA NDA Panel, 2023]. Collectively, these reports concluded that such products are safe for human consumption when cultivated in controlled conditions that minimize contamination, although attention should be paid to manganese intake. Based on these evaluations, fresh *W. arrhiza* and/or *W. globosa* plants have been authorized for marketing in the EU since 2021 under Commission Implementing Regulation (EU) 2021/2191, and in April 2024, the European Commission approved placing a protein concentrate from *L. gibba* and *L. minor* on the market.

In the US, the US Food and Drug Administration (FDA) has granted GRAS (Generally Recognized as Safe) status to some duckweed products, meaning they are considered safe for consumption as food ingredients. These include products derived from *Lemna* and *Wolffia* species. Plant powders derived from multiple *Lemnoideae* species, including polygenus (*Wolffia*, *Lemna,* and *Spirodella*), were granted GRAS status by the FDA in 2018. Today, duckweed is increasingly being considered in the Western diet as a sustainable alternative protein source that can replace meat while being healthier and more affordable [80]. In 2017, LENTEIN Complete and Degreened LENTEIN Complete from the Parabel company, which are made from duckweed protein, were deemed GRAS when used as nutrients in commercial food products (up to a maximum of 24 g per serving).

In Asia, especially in the Southeast Asian regions, duckweed has been officially consumed for over 25 years. It is considered safe for humans, with GRAS status, confirming its use in, among others, edible vaccines and functional foods for pharmaceutical applications. Unlike microalgae, duckweed is less likely to encounter regulatory barriers. In addition, one species of duckweed (*Spirodela polyrhiza*) is used to treat urticaria, acute nephritis, influenza, and inflammation in Japan, Korea, and China [78].

### 4.2. Potential Dangers Associated with Accumulation of Pollutants in Duckweed

The safety of consuming fresh *Wolffia arrhiza* and *W. globosa* plants is strictly dependent on the quality of the aquatic environment in which they are grown. These species exhibit the ability to accumulate arsenic and heavy metals, such as lead, cadmium, and chromium, as well as toxins, including cyanotoxins, e.g., microcystins. After death, these plants undergo rapid mineralization, which contributes to the intensive exchange of dissolved organic and inorganic substances, thus stimulating the development of bacteria and phytoplankton [84,85,86,87]. Heavy metals and microcystins may accumulate in the growing medium or originate from the use of fertilizers in *Wolffia* cultivation, which may pose a risk to their safety of consumption (Table 3).

The European Commission has also introduced general criteria for microbiological contamination. In the case of *Wolfia* used as a fresh product with a high moisture content, the EFSA has proposed reducing the maximum total microbial count from 10,000 to 1000 cfu/g. As regards the limit for *E. coli*, attention is drawn to the limit set in EC Regulation 2073/20056 for “cut fruit and vegetables (ready-to-eat)”, which is below 100 cfu/g.

The limit for *Enterobacteriaceae* and *E. coli* has been adjusted to 100 cfu/g, and the parameter coliforms has been removed, as they are included in the *Enterobacteriaceae* family. With regard to *Salmonella* spp. and *Listeria monocytogenes*, the EFSA indicated that, in accordance with EC Regulation 2073/2005, these pathogens cannot be detected in 25 g of product in the case of “cut fruit and vegetables (ready-to-eat)” or other “ready-to-eat foods” that may support the growth of *L. monocytogenes*, with the exception of products intended for infants and for special medical purposes. Therefore, the limit for *Salmonella* and *L. monocytogenes* is set at “not detectable in 25 g”. Fresh plants of *Wolffia arrhiza* and *W. globosa* can be placed on the market provided they meet these safety requirements. In addition to the risk of contaminant accumulation and microbiological contamination, the potential allergenicity of duckweed is also a significant safety risk. The EFSA evaluated the form and intake levels of *Lemnoideae*, as described by the company applying for its introduction into the EU. Based on these data, the EFSA concluded that the proposed consumption raises no safety concerns and that duckweed can be consumed by the general population as a vegetable for side dishes or as an ingredient in savory and meat-based meals, in typical amounts ranging from 100 g to 300 g of fresh weight per day. No information was provided regarding handling or preparation of the plants, nor were any specific precautions or restrictions on use indicated [58].

## 5. Potential Use in Targeted Nutrition

In the face of the world population’s constant growth, increasing globalization, and challenges related to providing food for everyone, it is necessary to seek new solutions in the field of its production. “New food” should be characterized by the greatest possible quality, including high nutritional value, and, at the same time, contribute to reducing food shortages in the world. Moreover, it should not only meet the energy needs of the body but also play an important role in disease prevention and support human growth as well as development. Additionally, in a world with changing climate conditions, conventional land crops of rice, soy, wheat, or corn, which require large land availability and significant inputs of fresh water and energy, are becoming less and less sustainable [88]. Therefore, alternative food sources are sought that will allow these requirements to be met. Eastern cuisine, in which plants from the *Lemnoideae* family play an important role, is becoming increasingly popular in Western countries. These plants are not only part of the traditional diet but are also valued for their numerous nutritional and biologically active properties [1]. In Table 4, an overview is presented of literature data on the active properties of selected plants from the *Lemnoideae* family. The studies summarized in the table indicate that the health-promoting properties of duckweed have been evaluated in diverse experimental models—ranging from in vitro analyses, through animal experiments, on to clinical trials involving humans. Laboratory assays (DPPH, FRAP, ABTS, TPC, and TFC determinations) confirmed the strong antioxidant activity of *Wolffia globosa*, *Wolffia arrhiza,* and *Lemna minor*, which is attributed to their high content of phenols, flavonoids, and carotenoids. Protein hydrolysates of duckweed exhibited strong inhibitory activity against the angiotensin-converting enzyme (ACE), highlighting their antihypertensive potential, as well as activity against other metabolic enzymes, suggesting a broad spectrum of bioactive effects. Extracts of *Lemna minor* demonstrated antibacterial activity against numerous Gram-positive and -negative bacterial strains as well as yeasts, while simulated digestion of *Wolffia globosa* confirmed its prebiotic properties. In animal feeding experiments, supplementation of fish diets with *Lemna minor* increased the content of long-chain omega-3 fatty acids (LC-PUFA), demonstrating the potential of this plant as a dietary component enriching the lipid profile. In clinical studies, consumption of *Wolffia globosa* (Mankai^®^) preparations resulted in reduced postprandial glycemia and glycemic index in both healthy individuals and patients with type 2 diabetes. Moreover, iron derived from this plant was shown to be bioavailable and may be effective in the prevention and treatment of iron deficiency anemia. There is also evidence suggesting the beneficial influence of duckweed on blood pressure and endothelial function, making it a promising subject for further cardioprotective research.

**Table 4:** Potential health benefits of bioactive compounds found in duckweed.

Effect	Species	Methodology	Outputs	Reference
Antioxidant activity	*Wolfia globosa*	DPPH Frap, Total phenolic content (TPC), Total Carotenoid content, Vitamin C content	*Wolffia globosa* exhibits strong antioxidant activity due to its high content of phenols, flavonoids, carotenoids, and vitamin C	[89]
	*Spirodela punctata, Lemna gibba,* and *Spirodela polyrhiza*, *Wolffia borealis*, and *Wolffiella caudata*	HPLC-DAD-ESI-MS/MS analysis of phenolic compounds, DPPH	DPPH free radical scavenging capacity: IC50 higher in species of the *Lemnoideae* subfamily, excluding *S. punctata*, compared to Wolffia and *Wolffiella*	[90]
	*Wolfia arrhiza*	TPC-Folin–Ciocalteu, Total Flavonoid Content (TFC)-Rutin equivalent, DPPH, FRAP	TPC significantly higher than in other plant materials. TFC significantly higher than in most edible vegetables and fruits High antioxidant activity	[31]
	*Lemna minor*	TPC-Folin–Ciocalteu, TFC-quercetin equivalent, Total antioxidant activity determination by ferric thiocyanate method, Total reduction capability, DPPH, ABTS	*Lemna minor* is effective antioxidant and antiradical in different in vitro assays, including reducing power, DPPH radical, ABTS•+ radical, and superoxide anion radical scavenging	[91]
	*Wolffia globosa*	TPC-Folin–Ciocalteu, TFC-quercetin equivalent, Total Anthocyanin Content (TAC)	Presence of compounds with antioxidant properties was found; it was also found that duckweed may have an effect on blood pressure and endothelial function in humans	[77]
Anti-diabetic, glucose-modeling, and cardioprophylactic effects	*Wolffia globosa*, Mankai^®^	Assessment of postprandial glycemia and glycemic index of product	Reduction in postprandial glucose and glycemic index after consuming duckweed	[92]
	*Wolffia globosa*, Mankai^®^	Assessment of postprandial glycemia in patients with type 2 diabetes	Reduction in postprandial glucose	[93]
	*Lemna minor*	*Lemna minor* consumption and evaluating level of fatty acids in products	Presence of duckweed in fish diet increased proportion of long-chain omega-3 fatty acids (LC-PUFA)	[94]
	*Wolffia globosa*, Mankai^®^	Evaluating level of iron in blood after consuming *Wolffia globosa*	Iron from duckweed is bioavailable and may be effective in treating iron deficiency-related anemia	[95]
Antihypertensive activity	Duckweed Powder	Enzymatic hydrolysis, fractionation, analyses performed: degree of hydrolysis (DH)—measured with OPA method; Protein/Peptide Content and Recovery Yields—via Dumas combustion method; Peptide Identification—by RP-UPLC-MS/MS (485 sequences identified); Total Phenolic Content (TPC)—Folin–Ciocalteu assay; Antihypertensive Activity—evaluated in vitro via ACE-inhibition spectrophotometric assay (IC50 determination)	Results indicate that enzymatic hydrolysis of duckweed proteins makes it possible to obtain bioactive fractions with antihypertensive potential, which positions duckweed as a sustainable source of health-promoting compounds	[21]
	Duckweed Powder	Enzymatic hydrolysis, fractionation. Analyses included UPLC-MS/MS: Ultra-Performance Liquid Chromatography with Tandem Mass Spectrometry; Protein content (Dumas): measured by combustion method; Bioactivity assays: ACE—Angiotensin-Converting Enzyme inhibition, DPP-IV—Dipeptidyl Peptidase IV inhibition, DPPH/TEAC—antioxidant capacity assays; Machine learning models: PLS-DA—Partial Least Squares Discriminant Analysis, QSAR—Quantitative Structure-Activity Relationship modeling	Highest activities in supernatants (DS). ACE inhibition IC50 = 0.07–0.08 mg/mL (FAR, TYY, IAI, Pep1: 13–40 μM); DPP-IV inhibition IC50 = 0.70 mg/mL (API, IPYDTQVK: 100–200 μM); antioxidant activity from peptides such as DYK, TYY, IGF. Some peptides showed multi-target effects (antihypertensive, antidiabetic, antioxidant).	[19]
Antimicrobial/antibacterial activity	*Lemna minor*	Disk-diffusion test	Duckweed extracts (both aqueous and ethanolic) have antibacterial activity against *Staphylococcus epidermidis*, *Staphylococcus saprophyticus*, *Staphylococcus warneri*, *Citrobacter freundii*, *Citrobacter koseri*, *Neisseria lactamica*, *Neisseria sicca*, *Micrococcus luteus*, *Bacillus cereus*, *Bacillus subtilis*, as well as *Streptococcus pneumoniae,* and show anti-candidophilic activity against *Candida parapsilosis* and *Candida glabrata*	[91]
	*Lemna minor*	Disk-diffusion test	Duckweed extracts have bacteriostatic and bactericidal effects against selected Gram-positive and -negative bacteria at MIC concentration = 1.8–2.0 mg/mL and MBC ≥ 2.0 mg/mL	[96]
Influence on intestinal microbiota (prebiotic) and anti-inflammatory effect	*Wolffia globosa*	Digestibility of *Wolffia globosa* in simulated human GI tract	*Wolffia* extracts demonstrate prebiotic potential	[28]
	*Lemnoideae*	Interleukin expression	Plants from this family have anti-inflammatory and anti-fibrotic effects	[97]

Notes: DPPH—2,2-diphenyl-1-picrylhydrazyl (assessment of antioxidant activity); TPC—Total phenolic content; HPLC-DAD-ESI-MS/MS—High-Performance Liquid Chromatography with Diode Array Detection and Electrospray Ionization Tandem Mass Spectrometry; TFC—Rutin equivalent; FRAP—Ferric Reducing Antioxidant Power; TFC-quercetin equivalent; TAC—Total Anthocyanin Content; ABTS*—2,2′-azino-bis(3-ethylbenzothiazoline-6-sulfonic acid), method measuring antioxidant activity by reduction in ABTSradical cation; RP-UPLC-MS/MS—Reversed-Phase Ultra-Performance Liquid Chromatography coupled with Tandem Mass Spectrometry; DH—Degree of Hydrolysis; Antihypertensive Activity—ACE-inhibition assay; DS—digest supernatants; ACE—Angiotensin-Converting Enzyme (IC_50_ = 0.07–0.08 mg/mL, 13–40 μM for FAR, TYY, IAI, Pep1); DPP-IV-—Dipeptidyl Peptidase IV (IC_50_ = 0.70 mg/mL, 100–200 μM for API, IPYDTQVK); Antioxidant activity—from peptides such as DYK, TYY, IGF; Some peptides exhibited multi-target effects (antihypertensive, antidiabetic, antioxidant).

The high antioxidant activity of duckweed species can be explained by their rich phytochemical composition, especially phenolic compounds and flavonoids, which are well-known free radical scavengers. Carotenoids and vitamin C further enhance this protective capacity by neutralizing reactive oxygen species. This synergistic profile of antioxidants may underlie not only the strong in vitro radical scavenging activity, but also the potential cardiovascular benefits observed in preliminary studies, such as effects on blood pressure and endothelial function. By reducing oxidative stress, duckweed-derived antioxidants could help protect vascular integrity and modulate mechanisms linked to hypertension and metabolic disorders.

Although the chemical composition of raw materials is crucial for their nutritional and health-promoting value, it is equally important to understand the changes that occur during food processing and digestion. Knowledge of the digestion process allows for the design of functional food products to be informed and tailored to the needs of specific consumer groups. This also applies to duckweed, which—despite growing interest—still remains poorly understood in terms of nutrient bioavailability and digestibility [28]. Supplementing this knowledge may be crucial to fully utilize its potential in human nutrition.

### 5.1. Nutrition for People with Special Protein Requirements

Providing adequate protein, including all essential amino acids, is crucial for proper growth, development, and maintaining health. Currently, almost half of the protein consumed comes from animal sources in developed countries [98]. However, the increasing challenges associated with producing animal protein—including its impact on the environment—are driving growing interest in alternative, plant-based protein sources.

Duckweed is a plant that is gaining recognition as a potential raw material in food production, especially in the nutrition of people with protein deficiencies or increased demand for this ingredient. Its protein has properties that allow it to partially or completely replace meat in the diet [78]. Moreover, the consumption of plant protein is associated with additional health benefits, such as increased intake of dietary fiber and bioactive plant compounds [99]. In comparison to animal protein, plant protein has been shown to provide a lower proportion of indispensable amino acids, bioavailable iron and zinc, and a smaller amount of vitamin B12 [100].

In studies, it has been shown that some species of duckweed can contain up to 45% protein on a dry matter basis, while maintaining a full amino acid profile in line with FAO standards [3]. Importantly, duckweed contains much more protein than cereals, making it a competitive source of this macronutrient [101]. It is worth emphasizing, however, that the protein content and its qualitative composition depend, to a large extent, on cultivation conditions, the selection of strains, and cloning methods [28,102]. Duckweed protein is a complete source of amino acids—it contains nine exogenous amino acids as well as six conditionally essential ones, and its corrected score is approximately 89% [95]. Its amino acid profile is comparable to proteins from other plant sources, such as soy [103] or lentils [104].

Compared to other plant proteins, it is distinguished by its high content of branched-chain amino acids (BCAA): isoleucine, leucine, and valine, which perform key metabolic functions—they are substrates for protein synthesis, signals stimulating this process, and can be used as a source of energy. For this reason, the presence of BCAA in duckweed may play an important role in the construction and regeneration of muscle tissue. It also has significance in the prevention or treatment of diseases such as sarcopenia [105,106,107].

In research on the subject, it was noted that BCAA supplementation leads to a significant increase in the thickness and strength of the quadriceps femoris muscle, which further confirms the potential of duckweed protein in the diet of physically active individuals [105].

In research on the extraction of protein from duckweed conducted by Dhamaratana, Methacanon and Charoensiddhi [28], it has been observed that the majority of proteins present in this plant are structural. The obtained protein isolate was characterized by a high protein content (66%), while, at the same time, low carbohydrate (11%) and ash contents (3%). Importantly, it also had a significant amount of phenolic compounds—26.60 mg GAE/g dry mass, which exceeded the levels recorded in many plants with known health-promoting properties, such as blueberries (25.92 mg GAE/g DW), olives (23.52 mg GAE/g DW), or parsley (6.94 mg GAE/g DW) [108]. The main protein found in duckweed is ribulose-1,5-bisphosphate carboxylase/oxygenase (RuBisCO), which provides a full set of amino acids essential for the human body [51]. Due to its high nutritional value, good in vitro digestibility, and lack of allergenic properties, the RuBisCO protein is considered a promising ingredient of functional foods [109]. Kawamata et al. [110] found that the protein did not show signs of toxicity.

Moreover, its technological properties make it a suitable substitute for egg whites, especially in industrial applications [111,112]. Liu, Wang, Huang, Cheng, Gan, Liu, Wu, Li, Peng and Geng [112] observed that RuBisCO exhibited similar denaturing properties as ovotransferrin, one of the main components of egg whites.

The assessment of protein quality in the context of nutrition for different population groups takes not only its amino acid composition into account, but also its digestibility, which largely determines the bioavailability of nutrients. In studies, it has been shown that the digestibility of duckweed protein exceeds that of typical plant proteins [11]. Similarly, Dhamaratana, Methacanon, and Charoensiddhi [8] observed that the digestibility of the extracted protein fraction does not differ significantly from that of the whole plant. According to these authors, duckweed protein has the potential to serve as a functional food ingredient that supports gut health and modulates gut microbiota. Moreover, in the studies by Kaplan et al. [95] and Sela et al. [113], it was demonstrated that duckweed protein is characterized by a complete profile of essential amino acids and high bioavailability, supporting its efficient absorption in both humans and animals. Duckweed can be a valuable dietary component not only for people with protein deficiencies, but also for vegans and vegetarians, who are particularly susceptible to deficiencies of certain micronutrients. In addition to the high content of complete protein, some species of duckweed show potential as a plant source of vitamin B12, which is usually found only in animal products. Studies conducted on the species *Wolffia globosa* (Mankai) [14,113] found significant amounts of vitamin B12 in a biologically active form. Moreover, in clinical studies, it has been shown that regular consumption of products containing *W. globosa* significantly increases the level of vitamin B12 in the blood serum of vegans, suggesting good bioavailability of this ingredient. These results further indicate that *Lemnoideae* can be not only a source of high-quality protein, but also a valuable supplement to a plant-based diet with key micronutrients, the deficiencies of which are typical in vegetarian and vegan diets [113].

### 5.2. Nutrition for People with Diabetes

The pathogenesis of type 2 diabetes largely depends on lifestyle, primarily including diet and the quality of consumed carbohydrates. In numerous studies, the beneficial effect has been confirmed of dietary fiber on lowering the glycemic index of meals, improving insulin sensitivity among people with type 2 diabetes and impaired glucose tolerance. As a result, increased fiber consumption may contribute to reducing the risk of type 2 diabetes and cardiovascular diseases. Particular importance is attributed to the soluble fraction—i.e., pectin—which, by forming gels, increases the viscosity of food, slows down digestion, and limits postprandial increases in blood glucose concentration [114]. In research, it has been shown that supplementation with glucans and glucomannans is an effective strategy for managing glycemia in people with T2DM [115,116]. In this context, plants of the *Lemna* genus, which are characterized by a high content of dietary fiber, seem to be particularly interesting [28]. Duckweed contains 23 to 25% of dietary fiber, including a unique pectin fraction—that is, apiogalacturonan—which has gelling properties [117]. Despite the scarce research on the soluble fiber content of duckweed, estimates suggest a range of 2–17% on a dry basis [28,118].

Due to high dietary fiber content (including the soluble fraction) and a significant share of protein, the consumption of plants from the *Lemnoideae* subfamily may have a beneficial effect on carbohydrate metabolism parameters such as post-prandial glycemia, glycemic index of meals, and tissue sensitivity to insulin. Moreover, the higher satiating power of duckweed compared to conventional foods can be attributed to its higher dietary fiber content [92]. Also noteworthy is the presence of numerous polyphenolic compounds, which have been shown to affect glucose metabolism. They act, among others, by inhibiting glucose absorption in the intestines, lowering fasting insulin levels, and reducing insulin resistance [92,119]. For this reason, *Lemnoideae* is a promising component of the diet for people struggling with type 2 diabetes or insulin resistance. The clinical effects of dietary polyphenols are not significant enough to recommend them for people with diabetes [120].

In the study conducted by Zelicha, Kaplan, Yaskolka Meir, Tsaban, Rinott, Shelef, Tirosh, Brikner, Pupkin and Qi [92], the authors evaluated postprandial and nocturnal glycemic response after consuming a “green smoothie” containing 25 g of duckweed compared to a yogurt-based one. The study involved adults with abdominal obesity. The results allowed us to indicate that consuming the smoothie with duckweed led to lower postprandial glycemic peaks (Δ = 13.4 ± 9.2 mg/dL) compared to the yogurt smoothie (Δ = 19.3 ± 15.1 mg/dL). Overall, lower postprandial blood glucose levels were also observed in the case of the smoothie with the addition of duckweed. In this study, confirmation was provided that duckweed can have a beneficial effect on glycemic response. This effect may be related not only to the high fiber content but also to the presence of polyphenols, which have been shown in other studies to reduce postprandial glycemic peaks and lower the glycemic index of products [92,121,122]. Although the effect is less than that of whey protein, duckweed protein also reduces the postprandial glycemic response [16].

In metanalyses of interventional studies, it has been confirmed that even moderate reductions in postprandial glycemia may be clinically relevant in the long term. Ouyang et al. [123] demonstrated that low-glycemic index diets reduced HbA1c by approximately 0.3% and fasting glucose by 0.4 mmol/L, with similar findings reported by Zafar et al. [124]. Earlier, Barclay et al. [122] showed that lower dietary glycemic index and load were associated with a reduced risk of type 2 diabetes and cardiovascular disease. These results suggest that the moderate reduction in postprandial glycemia observed in the trial by Zelicha et al. [92] may have meaningful clinical implications if maintained through regular consumption.

There have also been numerous studies on Mankai^®^—a cultivar of *Wolffia globosa*. In them, it has been suggested that a diet enriched with this plant may contribute to weight loss, improved glucose homeostasis, and beneficial changes in plasma lipid profile [93,125]. In the research carried out by Tsaban, Aharon-Hananel, Shalem, Zelicha, Yaskolka Meir, Pachter, Goldberg, Kamer, Alufer and Stampfer [93], the potential benefits of Mankai^®^ have also been demonstrated in postprandial glycemic control among patients with type 2 diabetes. In a randomized crossover study on 45 individuals with stable type 2 diabetes, it was found that consumption of a beverage containing Mankai^®^ after dinner led to significantly lower postprandial glycemia peaks and a shorter time to reach them compared to a yogurt-based drink. Similar conclusions were reached in the DIREDT PLUS trial, where the effects of the Mediterranean diet enriched with Mankai were examined on metabolic parameters in people with abdominal obesity or dyslipidemia. The 18-month study allowed for to demonstrate of beneficial effects of Mankai on glycemia control and no adverse effects associated with long-term use. In addition, consumption of a Mediterranean diet enriched with Mankai has been shown to have a positive influence on cardiometabolic risk and gut bacteria [92].

In light of the research conducted so far, duckweed seems to be a promising ingredient in the production of functional foods, especially as an alternative source of carbohydrates for people with glucose-insulin metabolism disorders. The results of the research allow us to suggest its beneficial effect on glycemic parameters, but their number is still limited, which emphasizes the need for further, well-designed clinical studies. Undoubtedly, this plant can be a valuable element of the diet for diabetics, not only as a source of high-quality fiber and protein, but also due to the presence of bioactive micronutrients found in it.

### 5.3. Potential Use of Lemnoideae in Supporting Cardiovascular Well-Being

In numerous studies, it has been confirmed that lipids are not only a source of energy but also play a crucial role in key physiological processes. They serve as structural components of cell membranes, act as precursors of eicosanoids, and regulate blood pressure as well as inflammatory responses. From a nutritional perspective, essential polyunsaturated fatty acids (PUFAs), particularly omega-3 fatty acids, are of special importance [126]. Their presence in the diet has been associated with a wide range of health benefits, including blood pressure reduction, lowering the risk of coronary heart disease and arrhythmias, as well as beneficial effects in neurodegenerative disorders, depression, and age-related macular degeneration [127].

In this context, duckweed emerges as a plant of interest. Although it contains relatively low total lipid levels, the fatty acids present are of high quality, with linoleic and α-linolenic acids as the main components [78]. Notably, duckweed is distinguished by its relatively high omega-3 content and a favorable omega-6 to omega-3 (n6/n3) ratio of approximately 0.5 [9]. However, it is important to recognize that the omega-6:omega-3 ratio remains a subject of scientific debate. As highlighted by Harris [128], this index should not be regarded as a stand-alone marker of health outcomes, since evidence primarily reflects correlations rather than causal relationships. Moreover, recent prospective data indicate no significant association between the dietary n6/n3 ratio and the risk of hypertension [129]. Therefore, when evaluating the lipid quality of duckweed, emphasis should be placed not only on the ratio itself but also on the absolute intake and bioavailability of omega-3 fatty acids, which may play a decisive role in mediating potential health benefits.

It is worth emphasizing that the potential of duckweed as a source of beneficial fatty acids is also reflected in model studies involving animals. Attempts have been made to use this plant as a component of feed in aquaculture. In studies, it has been exhibited that the addition of duckweed to the diet of tilapia resulted in a significant reduction in the total lipid content in the body and an increase in the level of long-chain omega-3 fatty acids (LC-PUFA) in their muscles [94]. Importantly, this effect may also be beneficial for humans—the authors indicate that including 15–20% of duckweed in feed improves the profile of omega-3 fatty acids in the meat of fish intended for consumption. Similar results were obtained in studies involving carp fry—also in this case, the diet with the addition of duckweed caused an increase in the content of polyunsaturated *n*-3 fatty acids in fish tissues [130]. In studies on the mass production of *Lemna minor* (*Lemnaceae*), fatty acid composition was determined, in which polyunsaturated fatty acids (PUFAs) predominated—accounting for 60–63% of total fatty acids—mainly α-linolenic acid (ALA, 18:3*n*-3) at approximately 41–47% and linoleic acid (LA, 18:2*n*-6) at 17–18% [51]. In addition to these nutritional benefits, in recent studies, it has been demonstrated that duckweed protein hydrolysates are a promising source of bioactive peptides with antihypertensive (ACE-inhibitory), antidiabetic (DPP-IV inhibitory), and antioxidant activities, identified and validated both experimentally and through machine-learning-assisted analyses [19,21].

In addition to its beneficial lipid profile, duckweed also has antioxidant properties, which may be important in the context of preventing cardiovascular diseases. In the research by Monthakantirat, Chulikhit, Maneenet, Khamphukdee, Chotritthirong, Limsakul, Punya, Turapra, Boonyarat and Daodee [77], it was demonstrated that this plant is a rich source of compounds with antioxidant properties, i.e., beta-carotene, ferulic acid, luteolin-7-O-β-D-glucoside, and kaempferol. These compounds can reduce oxidative stress by neutralizing free radicals, which help protect endothelial cells and support the proper functioning of blood vessels.

Given the presence of antioxidant compounds, omega-3 acids, and fiber, duckweed can be successfully included in cardioprotective diets, such as DASH (Dietary Approaches to Stop Hypertension). Its nutritional composition not only helps control lipid and glycemic profiles, but also reduces inflammation and protects blood vessels, making it a promising ingredient in the nutrition of people with cardiovascular diseases.

### 5.4. Nutrition for Immunosuppressed People

Plants from the *Lemnoideae* subfamily, especially *Lemna minor* and *Wolffia globosa*, have been used for years in traditional Asian medicine, homeopathy, and as additives to foods [131,132]. Despite limited modern scientific research, the plants are known for their analgesic, antipyretic, anti-inflammatory, astringent, and antipruritic effects, and can also be applied externally to treat eczema, acne, wounds, and insect bites [91,133]. In natural medicine, duckweed extracts are used, among others, in the treatment of allergy-induced asthma and rhinitis [131].

The antimicrobial properties of duckweed have been documented in numerous in vitro studies. Gulcin, Kirecci, Akkemik, Topal and Hisar [91] demonstrated that water and ethanol extracts of *Lemna minor* are active against many Gram-positive and -negative bacteria, such as: *Staphylococcus epidermidis*, *Citrobacter freundii*, *Neisseria lactamica*, *Bacillus subtilis*, *Streptococcus pneumoniae*, and yeasts: *Candida parapsilosis* and *Candida glabrata*. Tan, Hamdan, Mohamed, Choong, Chan and Lee [96] also confirmed the bactericidal activity of methanol extracts at concentrations of MIC = 1.8–2.0 mg/mL and MBC ≥ 2.0 mg/mL. In the research conducted by Dhamaratana, Methacanon and Charoensiddhi [28], it was shown that the consumption of duckweed promoted the growth of beneficial gut microorganisms, such as *Megamonas*, *Bifidobacterium*, *Phocaeicola*, *Bacteroides,* and *Blautia*. At the same time, increased synthesis of short-chain fatty acids (SCFA) was observed. Similar results were also obtained by Diotallevi, Gaudioso, Fava, Angeli, Lotti, Vrhovsek, Rinott, Shai, Gobbetti and Tuohy [125], confirming the beneficial effects of duckweed on the composition of the intestinal microbiota and SCFA metabolism. The antimicrobial effect of duckweed may therefore support the body in fighting bacterial and fungal infections.

Plants from the *Lemnoideae* subfamily also exhibit potential anti-inflammatory and immunostimulating properties, which result from the richness of bioactive compounds present in them. *Lemnoideae* contains numerous phenols, including flavonoids, tannins, and phenolic acids, as well as terpenoids and alkaloids—compounds with known anti-inflammatory effects [131,134,135]. Moreover, studies on animal and cell models allow us to confirm that some compounds isolated from these plants can affect the immune system, including by modulating the activity of macrophages and regulating the secretion of pro- and anti-inflammatory mediators [136,137,138]. In turn, Karamalakova, Stefanov, Georgieva and Nikolova [97], in their research on a mouse model with idiopathic pulmonary fibrosis, showed that supplementation with duckweed reduces oxidative stress and lowers the expression of proinflammatory cytokines. It is worth noting, however, not all studies confirmed the immunomodulatory effect of *Lemnoideae*. Cardoso, et al. [139] did not observe immunostimulatory effects of *Lemna minor* extracts on human immune cells assessed in vitro. At the same time, they noted that these extracts did not exhibit cytotoxicity or induce apoptosis, which indicates their safety of use.

Research on the potential of *Lemnoideae* in cancer prevention and immune support is still in its early stages and mostly involves in vitro models or animal trials. However, due to the presence of numerous bioactive compounds such as flavonoids, phenolic acid, quercetin, or beta-carotene, plants from this family exhibit antioxidant, anti-inflammatory, and immunomodulatory properties. These compounds can affect the activation of immune cells and regulate inflammatory response, which are important both in the context of strengthening immunity and cancer prevention. For example, quercetin present in *Lemnoideae* can inhibit the proinflammatory NF-κB pathway and activate the protective Nrf2/HO-1 pathway, showing potential anti-cancer effects [140]. What is more, the presence of fiber and prebiotic compounds in duckweed may support immunity by favorably modulating the gut microbiota, which also affects immune balance [28,139].

The anti-cancer activity of *Lemnoideae* is also confirmed by the results of modeling studies. Yiyit Doğan [141] demonstrated that extracts of duckweed exhibited strong cytotoxicity (59–79%) against osteosarcoma cells (Saos-2), while having no toxic effect on healthy human bone cells (hFOB). RT-PCR research allowed us to indicate that the extract induces apoptosis via a mitochondrial pathway regulated by the ratio of bax/bcl-2 gene expression. Similar results were obtained in the study carried out by Siriwata [42] in which protein hydrolysates from duckweed were shown to inhibit the proliferation of ovarian cancer cells. This effect is attributed to the presence of bioactive peptides that may have anti-cancer properties.

*Lemnoideae* has multifaceted health-promoting effects, including antimicrobial, anti-inflammatory, antioxidant, and anti-cancer properties. It can provide valuable support in the nutrition of individuals with weakened immune systems and in the prevention of cancer. Despite the promising results of in vitro and animal model trials [142], further clinical studies are necessary to fully confirm the efficacy and safety of *Lemnoideae* in therapeutic nutrition.

### 5.5. Potential of Lemnoideae as Food Source for Astronauts

The term “space food” refers to specially designed meals and snacks that astronauts eat during their missions in space. A balanced diet, consisting of micro- and macro-nutrients, is essential during spaceflight. A balanced intake of carbohydrates is vital for ensuring adequate energy, for proteins to maintain muscle mass, and fats to provide concentrated energy. Appropriate amounts of vitamins and minerals are also crucial, with particular emphasis on calcium for bone health, which is associated with the stresses experienced by astronauts. Maintaining optimal hydration is crucial for astronauts in space; thus, proper space food should prevent dehydration, support physiological functions, and help remove waste [143,144]. Astronauts require a certain amount of calories to support their daily activities and metabolic processes, with the average energy requirement in space being around 2500–3500 kcal/day. The variety and taste of space food are also considered to make meals enjoyable for astronauts. Providing familiar tastes and textures can help boost morale and maintain mental well-being during space missions [144]. Space food must also be lightweight, compact, and easy to store. It must also withstand the extreme temperatures and lack of gravity in space. The food is also designed to be consumed without creating crumbs that could float around in the spacecraft and potentially damage equipment [143].

Space farming requires plants that can thrive in limited resources of land, water, and energy. These plants must be able to process nutrients in a closed system and survive in microgravity. The most suitable plants for space farming should grow quickly, produce a large amount of biomass and protein, generate oxygen, and also help convert waste into useful nutrients [88]. As a promising potential source of nutrients for astronauts, due to their compact size, high nutrient density, high protein content, and potential for cultivation on space stations, aquatic plants—mainly from the *Wolfi* species—have been considered. Their high adaptability to cultivation in a controlled environment makes them promising candidates for space agriculture, potentially supporting long-term missions by providing food, recycling waste, and maintaining air quality [145]. Various species of duckweed have been described as very well adapted to produce large amounts of biomass with high nutrient density in the harsh conditions of space. Among them, excellent candidates for space cultivation are species such as *Wolffia globosa*, *W. arrhiza*, and *W. australiana*, which grow very quickly, also in unfavorable and extreme conditions, and are characterized by high nutrient density [145]. Additionally, *Wolffia* species have the ability to purify wastewater by removing nitrogen (>5 mg N/L), phosphorus (>1 mg P/L), and organic pollutants [146]. *Wolfia globosa* can survive in environments with very low oxygen and nutrients, so it can be stored for long periods of time, after which it can grow in closed systems during space travel [147,148]. Additionally, it has shown stable growth in microgravity and hypergravity conditions, making it a potential source of high-protein food during space missions [88]. *W. globosa* has also been successfully grown in Mars-like conditions, with low light and limited nutrients, and has performed well in bioreactors treating artificial sewage. Radiation tests conducted on *W. globosa* have shown minimal DNA damage, confirming its ability to survive in space and the stability of the species [149].

*Wolffia arrhiza* can survive extreme conditions and environmental stress because of its ability to form turions—resting bodies that allow it to survive in unfavorable conditions [150]. This species not only exhibits a high tolerance to harsh conditions, but also maintains growth and intensive photosynthesis in high salinity and low oxygen concentrations, making it a promising organism for use in wastewater recycling in space conditions [151]. *W. arrhiza* has additionally been shown to grow well in nutrient-rich environments with high CO_2_ concentrations (approx. 800 ppm), especially in closed systems such as sewage treatment plants. In such conditions, the plant can double its mass in just 1.4–2.1 days, while contributing to oxygen production [34,145]. Similarly, *Wolfia australiana* can survive in low-nutrient and reduced-gravity environments, making it suitable for nutrient recycling and oxygen production at space stations [152]. *W. australiana* also demonstrates high biomass production even when grown in lunar soil simulators, proving its suitability for cultivation on other planets [152,153].

Due to their low resource requirements, rapid growth, efficient CO_2_ uptake, and ability to generate oxygen, *Wolffia globosa*, *W. arrhiza,* and *W. australiana* are considered promising species for space cultivation [154]. They grow faster, process nutrients better, and adapt more sufficiently to space conditions than traditional crops or microalgae, making them valuable components for future space missions and planetary settlements [88]. With further research and technological development, the *Wolffia* species could play a key role in sustainable space agriculture.

## 6. Consumer Perception, Applications, and Market Challenges

Although plants from the *Lemnoideae* subfamily are distinguished by their high protein content and favorable environmental characteristics, their wide introduction into the diet of consumers, especially in Western countries, may encounter numerous sensory and psychological barriers. Both organoleptic properties and mechanisms of perceiving nutritional novelties play a key role in the process of accepting this plant as a food ingredient. In recent studies, it has been confirmed that duckweed powders, despite their promising protein levels and micronutrient profile, show considerable variability in amino acid balance, mineral content, and volatile compounds that may influence consumer perception as well as acceptance [47,155]. According to De Beukelaar, Zeinstra, Mes and Fischer [101], Western consumers most often categorize duckweed as a vegetable. This plant is perceived as most suitable in the context of savory dishes, where the presence of green ingredients is expected, such as salads, soups or dinners. In turn, its use in sweet or dessert products reduces acceptance, even if the consumer receives information about its health and ecological values. Barriers to the acceptance of duckweed as a food and possible strategies to overcome them are presented in Table 5.

One of the key factors limiting consumers’ willingness to consume duckweed is the low awareness of it being a food ingredient. According to Onwezen, Bouwman, Reinders and Dagevos [156], familiarity with a product is one of the strongest predictors of its acceptance. Consumers have a natural tendency to prefer products that they are familiar with, both through previous taste experiences and their familiarity with a given cuisine or culture. Since duckweed is not widely recognized in Western countries, it arouses uncertainty and sometimes even reluctance. In addition, the natural habitat of duckweed also plays a major role; consumers may perceive the plant associated with stagnant waters as unappetizing or even dangerous. The sensory characteristics of duckweed may also constitute a barrier. The green color, earthy smell, or grassy aftertaste do not always match consumer expectations, especially in products such as bread, desserts, or snacks. At the same time, as shown in studies on the acceptance of microalgae, these products are better received when they are hidden in familiar culinary forms and associations (e.g., as part of a smoothie, stuffing or soup) [157]. According to Matos, et al. [158], despite their high nutritional value, aquatic plants often encounter resistance from consumers precisely because of their intense green color, specific taste, and “marine” smell. European consumers prefer products with microalgae when they are used in small quantities and have a neutral effect on the taste and appearance of the dish. Although duckweed is not a microalgae, it is characterized by a similarly intense color and the presence of chlorophyll, which may trigger comparable sensory and social reactions.

In order to increase the acceptance of duckweed products, it is therefore necessary to take not only the rational, but also the emotional and cultural aspects of food perception into account. Market success may depend on the way the product is presented (e.g., esthetic packaging, attractive branding), matching it to an appropriate culinary context, and building positive associations as well as consumer habits. Strategic communication, e.g., presenting duckweed as a “superfood” from Asia or “green protein of the future”, may help to overcome initial resistance and build interest among environmentally- and health-conscious consumers [101]. The use of *Wolffia globosa* in the context of functional food development has been subjected to detailed analysis in the research by On-Nom, et al. [159]. In their study, not only the nutritional value but also the sensory acceptance of a high-protein snack containing duckweed was assessed. In this trial, it was demonstrated that the use of 26% freeze-dried *W. globosa* powder, in combination with rice and tapioca flours, resulted in a product with high sensory acceptability. Although slightly lower texture and taste scores were noted compared to the control snack, the overall consumer rating remained at a level above six on a nine-point hedonic scale, indicating the market potential of such products. Importantly, despite the characteristic green color resulting from the chlorophyll content, the color did not have a negative impact on consumer acceptance. The results of this study allow us to confirm that a properly developed product containing duckweed can be both attractive in a sensory manner and also nutritionally complete.

In recent years, the growing interest in duckweed as an innovative food ingredient has resulted in a number of commercial and research initiatives. Companies such as Parabel, Hinoman, GreenOnyx, and Plantible are actively promoting duckweed, including *Wolffia globosa*, as a sustainable source of plant protein and micronutrients. At the same time, more and more chefs and home users are experimenting with the culinary potential of this plant, recognizing its neutral taste, attractive color, and the possibility of enriching dishes through its nutritional value [1]. *W. globosa* has already been used as an ingredient in various dishes: it is added to cocktails, soups, salads, pancakes, dumplings, and groats [95]. The market for finished products, with its inclusion, is also developing dynamically. For example, breads of intense green color, wraps, smoothies, green vegan cheesecakes as well as fusion cuisine dishes, such as sushi with *Wolffia*, pasta with scallops, dishes from fish and tofu, and vegetarian snacks, are being created (flowolffia.com; Holistic Chef Academy, 2025; Unilever Food Solutions, 2024).

Importantly, duckweed not only enriches food nutritionally, but can also affect its durability and technological properties. In the research by Yahaya, Hamdan, Zabidi, Mohamad, Suhaimi, Johari, Yahya and Yahya [78], the authors observed that adding 2% of duckweed powder to ice cream led to an increase in protein and fiber content without negatively affecting the microbiological quality of the product. In turn, Rocchetti, et al. [160] noticed that duckweed extract may exhibit pro-oxidant properties in beef burgers, which may extend shelf-life and improve lipid stability during storage. The potential of duckweed as a natural antioxidant has also been confirmed in the study by Gulcin, Kirecci, Akkemik, Topal and Hisar [91]. Water extracts from this plant showed high in vitro antioxidant activity, such as the ability to scavenge DPPH· and ABTS•+ radicals, metal chelating activity, and reducing power. A significant correlation was also observed between the content of phenolic compounds and total antioxidant activity. This may indicate the possibility of using duckweed as a preservative ingredient, delaying oxidation processes in food and thus supporting the maintenance of its quality and safety. Although research on the use of duckweed in food products is still ongoing, the results obtained to date are promising and indicate a wide spectrum of its functional properties. Thanks to the possibility of enriching food with protein, fiber, antioxidants, and minerals, as well as its impact on the durability of products, *Wolffia* spp. is gaining the title of the plant of the future in the context of both functional food and innovative food product design.

## 7. Challenges Regarding Bioavailability and Processing of Duckweed

Despite the high nutritional potential, the use of proteins and other bioactive compounds contained in duckweed may be limited by a number of factors. The most important include plant growth conditions, the structure and properties of cell walls, the presence of secondary metabolites (phenols: phenolic acids, flavonoids, stilbenes, lignans, and carotenoids: β-carotene, α-carotene, lutein), and antinutritional components (oxalic acid, protease inhibitors, tannins) [161]. These factors can affect both the digestibility and bioavailability of nutrients, i.e., their absorption and utilization by the body. In order to increase the availability of these compounds to the body, it may be necessary to use appropriate processing methods—physical, chemical, or biochemical—such as fermentation, cooking, grinding, and enzymatic treatment. Although some strategies (soaking, germination, fermentation, heat treatment debranning) for improving digestibility and reducing antinutritional factors in plant products have been described in the literature, there is still a lack of sufficient data regarding the effects of these treatments on duckweed [162].

Available reports are mostly limited to general processing approaches such as drying, milling, protein extraction, fermentation, or enzymatic hydrolysis, without providing detailed insights into how these methods specifically influence the nutritional composition and sensory quality of *Lemnoideae.* To date, in no comparative studies have these processing methods been systematically evaluated. Further research is needed on how specific technological processes and food preparation methods affect the nutritional value and sensory properties of duckweed and products containing it. To date, only a limited number of studies have been conducted on the digestibility (i.e., the amount of nutrients released during digestion) and bioavailability (i.e., the proportion of nutrients that are absorbed and used by the body) of compounds contained in duckweed [163,164]. An additional challenge is the high variability of the nutritional composition, which depends on the species and strain of the plant, environmental conditions, the time of harvest, and geographical location. Research on this variability is scarce, and its results are often difficult to generalize [1].

To fully exploit the potential of duckweed as a sustainable food or feed ingredient, comprehensive research is needed, covering nutritional, technological, and sensory aspects. This would include developing effective formulations, assessing consumer acceptability, and analyzing the impact of duckweed on the taste and smell of the final products. Such an interdisciplinary approach could contribute to promoting duckweed as a valuable, sustainable source of nutrients in global food systems.

## 8. Conclusions

Plants from the *Lemnoideae* subfamily, and especially representatives of the *Wolffia* genus, are an extremely promising source of plant protein, the potential of which in human nutrition remains untapped. The high content of full-value protein, favorable amino acid profile, presence of bioactive components, and good digestibility make duckweed a suitable component of functional food, especially within the context of so-called targeted nutrition. The possibility of using *Lemnoideae* in the diet of individuals with protein deficiencies, diabetes, and cardiovascular diseases, as well as in the prevention of cancer and supporting immunity, indicates a wide range of its applications. Moreover, *Lemnoideae* is characterized by a beneficial effect on the environment. Its cultivation does not require large spatial outlays or water resources, and the rapid increase in biomass makes it a sustainable alternative to traditional sources of animal protein. Their low allergenic potential, lack of raphides (in the case of *Wolffia*), as well as increasing availability and consumer acceptance, indicate the growing role of these plants in the future of the human diet. Despite numerous benefits, it is necessary to continue research on the safety of consuming *Lemnoideae*, especially in the context of their ability to accumulate heavy metals and microbiological contamination. This requires the development of rigorous quality standards for crops and methods of monitoring and controlling the content of undesirable substances. It should be noted that the current evidence base regarding *Lemnoideae* in human nutrition is still limited. Most conclusions are derived from short-term clinical trials or animal models, and there is a lack of long-term studies across diverse populations. Future research should, therefore, be focused on well-designed longitudinal interventions to confirm the efficacy and safety of *Lemnoideae* consumption in different target groups. It is equally important to develop methods of processing duckweed that would allow its nutritional and functional values to be preserved while ensuring attractive sensory properties. In the future, interdisciplinary clinical studies are desirable, including, among others, on the bioavailability of nutrients, effects on intestinal microbiota, and long-term safety of use. It is also worth developing directions for the use of *Lemnoideae* in the nutrition of astronauts, for individuals undergoing periods of convalescence, and in medical nutrition. Additional research challenges include the possibilities of metabolic engineering and selection of strains in terms of the content of selected nutrients (e.g., vitamin B12, EPA, DHA). In summary, *Lemnoideae*—, as a component of future functional foods—can play a key role in the global transformation of food systems.

## Figures and Tables

**Table 3:** Specifications proposed by EFSA for the marketing of Wolffia species intended for human consumption [83].

Heavy Metals	General Safety Limits
lead	<0.3 mg/kg
arsenic (inorganic)	<0.10 mg/kg
cadmium	<0.2 mg/kg
chromium	<1 mg/kg
mercury	<0.10 mg/kg
copper	<0.8 mg/kg
molybdenum	<0.3 mg/kg
zinc	<5 mg/kg
boron	<5 mg/kg
manganese	<6 mg/kg
cyanotoxins	microcystins: 0.006 μg/g fresh weight

**Table 5:** Barriers to acceptance of duckweed as food and strategies to overcome them.

Barrier	Description of Problem	Countermeasure Strategy
Green color and general appearance	Reminds me of algae, a pond, “something dirty”	Esthetic packaging, attractive branding (does not require the use of additional colorants, contains a natural colorant). In the future, ‘green protein’ or ‘superfood’ could be potentially authorized, provided that the necessary studies are conducted and confirmation is obtained from the relevant authorities
Unfamiliarity with product	Lack of trust, no cultural references	Educational campaigns, marketing based on health and environmental values
Taste and aroma	Bitter or grassy aftertaste	Fermentation, seasoning, combining with vegetables or known culinary ingredients
Context of meal	Not suitable for desserts and sweet dishes	Use in salads, soups, burgers—as added vegetable
Negative associations of “duckweed”	Reminds me of ducks, a pond, “weeds”	Using alternative names: “water lentils”, “green protein”, “*Lemna* blend”
Food neophobia	Fear of novelty, reluctance to experiment	Tastings, social media campaigns, presence in well-known brands
Safety and cleanliness concerns	Fear of water pollution, toxins	Information on controlled crops, quality certificates, purity tests
Lack of presence in Western cuisine	Lack of cultural familiarity with product	References to Asian cuisine (e.g., “Khai-nam”), incorporation into well-known fusion dishes

Source: own elaboration based on de Beukelaar et al. [101], Onwezen et al. [156], Olsen et al. [157].
